# In vivo imaging of early stages of rheumatoid arthritis by α5β1-integrin-targeted positron emission tomography

**DOI:** 10.1186/s13550-019-0541-6

**Published:** 2019-09-09

**Authors:** Johannes Notni, Florian T. Gassert, Katja Steiger, Peter Sommer, Wilko Weichert, Ernst J. Rummeny, Markus Schwaiger, Horst Kessler, Reinhard Meier, Melanie A. Kimm

**Affiliations:** 1 Institute of Pathology, Technische Universität München, Trogerstr. 18, 81675 Munich, Germany; 2 Department of Diagnostic and Interventional Radiology, Technische Universität München, Munich, Germany; 3 Department of Nuclear Medicine, Technische Universität München, Munich, Germany; 4 Institute for Advanced Study, Department of Chemistry, Technische Universität München, Garching, Germany; 5 German Cancer Consortium (DKTK), Partner Site Munich, Munich, Germany

**Keywords:** Rheumatoid arthritis, Positron emission tomography, Integrins, Animal models, Collagen-induced arthritis, Immunohistochemistry

## Abstract

**Background:**

Rheumatoid arthritis (RA) is one of the most common rheumatic diseases. Joint inflammation and pathological growth of joint cartilage cause swollen and painful joints, which severely diminishes the patients’ life quality. There is no causal treatment. Symptomatic therapies should start as early as possible to take maximal effect. Hence, diagnostic procedures capable of detecting affected joints before the onset of clinical symptoms are highly desirable. We explored the value of PET imaging of integrin subtypes αvβ3 and α5β1 for early detection of RA foci in collagen-induced arthritis (CIA) mouse models.

**Results:**

Development of RA in CIA mice was monitored by paw scoring, and αvβ3- and α5β1-integrin expression was quantified by μPET using ^68^Ga-Avebetrin and ^68^Ga-Aquibeprin. For consecutive sections of selected decalcified joints (knee, ankle), arthritic degeneration and integrin expression were assessed by MOVAT staining and β3/α5 immunohistochemistry (IHC), respectively. β3- and α5-IHC revealed elevated levels of both αvβ3- and α5β1-integrin in arthritic joints. Unlike αvβ3, α5β1 is strongly expressed in the proliferating synovial lining layer, which suggests that its presence is directly related to RA development. For mice with advanced RA (6 weeks after CIA), PET signals for α5β1-integrin were substantially stronger (> 300% of baseline) than that of αvβ3-integrin (< 200%). A longitudinal PET follow-up revealed that the manifestation of clinical symptoms of RA is preceded by upregulation of α5β1- but not of αvβ3-integrin.

**Conclusion:**

α5β1-integrin PET could add a new functional imaging aspect to the portfolio of RA diagnostics because it appears to be a sensitive biomarker for early RA development. We suggest α5β1-integrin PET as a valuable tool to achieve a higher precision for early diagnosis of RA, including initial staging, monitoring of the disease course, and drug treatment, and for planning of radiosynoviorthesis (RSO).

## Background

Rheumatoid arthritis (RA) [1] is one of the most common rheumatic diseases and affects between 0.3 and 1.0% of adults worldwide, with 5–50 new cases per 100,000 being diagnosed each year. RA is an inflammatory autoimmune disorder primarily affecting joints and is associated with clinical symptoms typical for inflammation, such as warm, swollen, and painful joints. RA is presumably caused by both genetic predisposition and environmental factors. In contrast to osteoarthritis (OA) which is a widespread, non-inflammatory degenerative joint disease characterized by a loss of cartilage, the RA-inducing autoimmune attack causes an abnormal growth of fibrovascular tissue on the surface of the joint cartilage, called pannus, but also an inflammation of the synovial membrane which leads to over-production of synovial fluid and a degeneration of the underlying bone. Currently, there is no causal treatment for RA. The common symptomatic therapies thus aim to reduce pain, inflammation, and progress of degeneration, in order to preserve the joint function and the life quality of patients as long as possible. The established long-term therapies rely on the systemic application of disease-modifying anti-rheumatic drugs (DMARDs), above all, the immunosuppressive agent methotrexate (a folate receptor inhibitor) [2], or biologicals, such as tumor necrosis factor α (TNF-α) inhibitors [3–5]. Such therapies are most successful when the treatment is started early and aggressively [6], which in consequence, highlights the paramount importance of an early diagnosis.

In the course of the disease, patients who do not sufficiently respond to drug treatment may undergo a synovectomy, that is, the surgical removal of the synovial membrane which represents the main area of inflammatory processes and disease progression, typically resulting in a reduction of the symptoms of inflammation. However, a comparable effect can be achieved by the metabolic inactivation of the synovium by means of radiosynoviorthesis (RSO, also termed radiosynovectomy), a non-invasive treatment of singular rheumatic joints. RSO as well as surgery can also be used early on to avoid the adverse effects of systemic medication. For RSO, formulations containing β^−^-emitting radionuclides with intermediate half-lives and high, intermediate, or low β^−^ energy, namely ^90^Y (*t*_½_ = 2.7 days, *E*_β_^max^ = 2.26 MeV), ^186^Re (*t*_½_ = 3.7 d, *E*_β_^max^ = 890 keV), or ^169^Er (*t*_½_ = 9.5 d, *E*_β_^max^ = 340 keV) are injected directly into the joint cavity of large (knee), medium-sized (shoulder, wrist, ankle), or small (finger, toe) joints, respectively. The strictly local β^−^ irradiation inactivates the inflammatory cells (osteoblasts and fibroblasts), whereupon the inflammation stops and disease progression ceases. Like DMARDs, the RSO is most successful when it is started as early as possible, ideally at a pre-symptomatic stage when the cellular signaling leading to inflammatory processes has just begun, but joints are still fully functional, i.e., degeneration and pannus formation have not started yet. Since RSO requires the identification of affected joints, planning of RSO treatment could be enhanced by the sensitive and selective functional imaging of biomarkers that are associated with the early development stages of RA in joints.

The *1987 Traditional Diagnostic Criteria of the American College of Rheumatology (ACR) for the Diagnosis of RA* and the *2010 ACR-EULAR classification criteria for rheumatoid arthritis* are mainly based on the clinical signs of RA and are widely recognized as the standard classification tree format with the highest sensitivity, specificity, predictive value, and accuracy [7–9]. This scheme is frequently supported by imaging techniques, such as radiography, ultrasound, and magnetic resonance imaging (MRI) [10, 11]. The latter facilitates an accurate diagnosis of early RA [12, 13], because it can be applied to confirm the presence of subclinical synovitis [14], to discover RA in clinical remission when there is no sign of swelling or tender joints [15] and to detect bone erosion with a higher sensitivity than plain radiography [16]. Furthermore, although it is not specific for RA, positron emission tomography (PET) with [^18^F]2-fluoro-2-deoxy-d-glucose (FGD) can reveal arthritic lesions, because the advanced inflammatory processes are generally associated with an elevated energy conversion and, consequently, a higher glucose consumption. Indeed, arthritic joints could be quite convincingly delineated by whole-body FDG-PET [17], and FGD-PET has been employed for the assessment of therapy response [18].

However, the sensitivity and also the prognostic and predictive value of the established imaging modalities are limited in view of their relative inability to specifically detect pre-symptomatic stages of RA, i.e., where morphology and functionality of joints is not yet affected but the cellular processes ultimately leading to joint destruction have already begun to change. In contrast to the abovementioned imaging approaches which are primarily determined to detect abnormal morphology or strongly altered metabolic rates, functional imaging of dedicated cellular biomarkers for RA could lead to a paradigm shift in the diagnosis and treatment of RA, particularly for patients with an elevated hereditary risk. Identification and functional imaging of such biomarkers is therefore highly important.

Since synovial angiogenesis is a key process in RA development [19, 20], it has been suggested as a specific target for imaging of early RA. Angiogenesis in RA is triggered by proliferating mesenchymal synovial cells as a response to their elevated levels of energy and oxygen consumption upon pannus formation. As a result, the cellular receptors involved in the angiogenesis signaling pathways are upregulated, among them the integrin subtypes αvβ3 and α5β1 which were found to be expressed in synovial endothelial cells and chondrocytes [21]. Hence, the radiolabelled derivatives of RGD peptides [22], which were successfully applied for αvβ3-integrin targeted PET in the context of cancer and cardiovascular diseases [23, 24], have also been evaluated in terms of their utility for RA imaging, but did not provide a substantial added value over and above FDG-PET [25]. Yet, the value of α5β1-integrin imaging [26] has not been evaluated in this context, although multiple evidence indicates that this subtype is a more reliable biomarker for angiogenesis [27] and it is upregulated earlier than αvβ3-integrin [28, 29]. Therefore, we aimed to elucidate the potential of a recently introduced α5β1-integrin targeting PET probe, ^68^Ga-Aquibeprin [30, 31], for the selective imaging of early angiogenesis in an established animal model for RA [32].

## Materials and methods

### Mouse model

Procedures involving laboratory mice and their care were conducted in conformity with institutional guidelines and with approval from the responsible local authorities. In total, 33 DBA/1JRj mice (7–8 weeks old) were obtained from Janvier Labs (France). Thirty mice were randomly categorized into 1 of 5 groups, each group comprising 6 mice. All five groups were subject to collagen-induced arthritis (CIA) according to a published standard protocol [32]. Briefly, DBA/1 mice were subcutaneously injected with 50 μg bovine type II collagen (Chondrex Inc., Redmont, USA) emulsified with equal amounts of Freund's complete adjuvant (Sigma Aldrich, Taufkirchen, Germany) into the base of the tail. Fourteen days later, mice were administered a booster injection at the base of the tail with Freud’s incomplete adjuvant (Sigma). The control group comprised 3 mice and was not subject to CIA. Groups 1 to 5 were subjected to PET imaging and MRI at time points of 1, 2, 3, 4, and 6 weeks after the beginning of RA development, respectively, and the control group after 6 weeks.

Paw scoring was done by monitoring paw swelling according to established standard criteria [32]. Briefly, each paw was measured for thickness, swelling, warmth, and redness and rated on a scale of 0–4. Score 0 was given when no evidence of erythema and swelling was observed, score 1 for erythema and mild swelling confined to the tarsals or ankle joint, score 2 for erythema and mild swelling extending from the ankle to the tarsals, score 3 for erythema and moderate swelling extending from the ankle to metatarsal joints, and score 4 for erythema and severe swelling encompassing the ankle, foot and digits, or ankylosis of the limb.

### MR imaging

MR images were acquired on Agilent/General Electric MR901 small animal MRI system. Mice were anesthetized with isoflurane and had a tail vein catheter implanted. Mice were placed on the scanner bed on their side, with the hind limb pulled out and placed with the ankle centered over a 3 cm flexible surface receive coil (RAPID Biomedical, Germany), held in place with adhesive tape. A 2D multislice spoiled gradient recalled echo sequence was used, with 6 slices of 0.5-mm slice thickness, TR 50 ms, flip angle 30°, receive bandwidth 31.25 kHz, and 80 averages. Scan timing, field of view, and acquisition matrix varied slightly depending on anatomical positioning but were approximately TE 2.05 ms, matrix size 128 × 64, and field of view 10 mm × 5 mm. Image slices were oriented approximately parallel to the bones of the leg, perpendicular to the surface of the paw. Baseline pre-injection and post-contrast injection scans were acquired in one session, approximately every 7 min, without moving the animal.

The MRI findings were evaluated by a radiologist (RM) who is experienced in imaging of arthritis. For evaluation of synovitis, the EULAR-OMERACT RAMRI score was applied [33]. Synovitis was scored on a scale of 0–3 in each joint, with a score of 0 representing no synovitis and scores of 1–3 representing mild, moderate, and severe synovitis, respectively. Synovitis was defined as an abnormal increase in signal intensity in the area of the synovium after Gadofluorine P injection (100 μmol/kg body weight, invivoContrast GmbH, Berlin, Germany) and an expanded synovial membrane.

### PET

μPET imaging was performed as described before [30]. Briefly, DBA mice, weighting 20–25 g at the time of final use for experiments, received injections of the radiopharmaceuticals for PET via tail vein catheters under isoflurane anesthesia. In order to achieve injections with constant amounts of biologically active compound, 500 pmol of the respective non-radioactive compound (Avebetrin or Aquibeprin, respectively) was added to each syringe and the actually injected total amount of cold mass calculated from syringe activities before and after injection as described previously [31]. For blockade, 30 nmol of non-radioactive compound was added. For cross-blockade, 30 nmol of the respective other non-radioactive compound were added, i.e., Avebetrin to a ^68^Ga-Aquibeprin injection or Aquibeprin to a ^68^Ga-Avebetrin injection. Non-blockade animals received 18 ± 3 MBq with molar activities of 38 ± 7 MBq/nmol, and compound mass doses were 480 ± 26 pmol. After injections, animals were allowed to wake up with access to food and water. PET was recorded under isoflurane anesthesia, 75 min p.i. for 20 min, on a Siemens Inveon small-animal PET system. Generally, ^68^Ga-Aquibeprin and ^68^Ga-Avebetrin scans were recorded virtually simultaneously in relation to disease progress, that is, performed in the same order (first ^68^Ga-Aquibeprin then ^68^Ga-Avebetrin) on the same day with a 6-h interval allowing for virtually complete decay of the first injected radiopharmaceutical (overall residual activity calculated to approx. 2%). Images were reconstructed as single frames using Siemens Inveon software, employing an ordered subset expectation maximum (OSEM) 3D algorithm without scatter and attenuation correction. All images shown are maximal intensity projections seen from dorsal, color scales ranging from 0 to 3% of injected dose per milliliter tissue.

Quantification of PET signals was performed by drawing spherical regions of interest (ROI) of standard volumes for knees (23.5 mm^3^) and ankles (3.4 mm^3^), centered to the respective voxels with maximal intensity. Paws were not analyzed because their small size and the resulting pronounced partial volume effect does not allow for reliable quantification of uptake. [^68^Ga features a high positron energy (E_β+,max_ = 1.9 MeV, E_β+,avg_ = 836 keV), resulting in a high inherent degree of blurring [34] The mean traveling distance of positrons in tissue prior to annihilation is 3–4 mm, which is exceeding the size of a mouse paw. Thus, the fraction of positrons leaving the paw tissue before local annihilation is considered too high to allow for a reliable quantification of the resulting PET signal, even more so because the stretched-out paws are surrounded by air and such positrons readily escape from the field-of-view of the scanner. Hence, despite being clearly recognizable in the images, any observed PET signal in paws is to be considered underestimated, which is however not relevant for prospective human application because of the much larger size of subjects.] ROI-based uptake values (% injected dose per mL tissue) were analyzed on a per-cohort basis and are expressed as average values ± standard deviation.

### Histopathology

After the last PET scan, mice were sacrificed, extremities fixed in 4 % PBS buffered formalin for 48 h, decalcified in Osteosoft (Merck, Darmstadt, Germany) for 4 weeks according to the manufacturer’s instructions, and embedded in paraffin. Sagittal sections of 4-μm thickness were cut, and consecutive sections stained with Movat pentachrome staining was performed using a staining kit (Morphisto, Frankfurt am Main, Germany). For immunohistochemistry (IHC), serial sections were stained using a Bond RXm system (Leica, Wetzlar, Germany, all reagents from Leica, unless otherwise indicated) with primary antibodies against α5-integrin (abcam, ab 108327, diluted 1:10.000) or β3-integrin (Cell Signaling, 13166S, diluted 1:300). Briefly, slides were deparaffinized using deparaffinization solution, pretreated with Epitope retrieval solution 2 (corresponding to EDTA buffer pH 8) for 30 min. Antibody binding was detected with a polymer refine detection kit without post-primary reagent and visualized with DAB as a dark brown precipitate. Slides were scanned (Leica AT2, Leica, Wetzlar, Germany) and evaluated using Imagescope Software (Leica, Wetzlar, Germany).

## Results

### Study outline

**Fig. 1:**
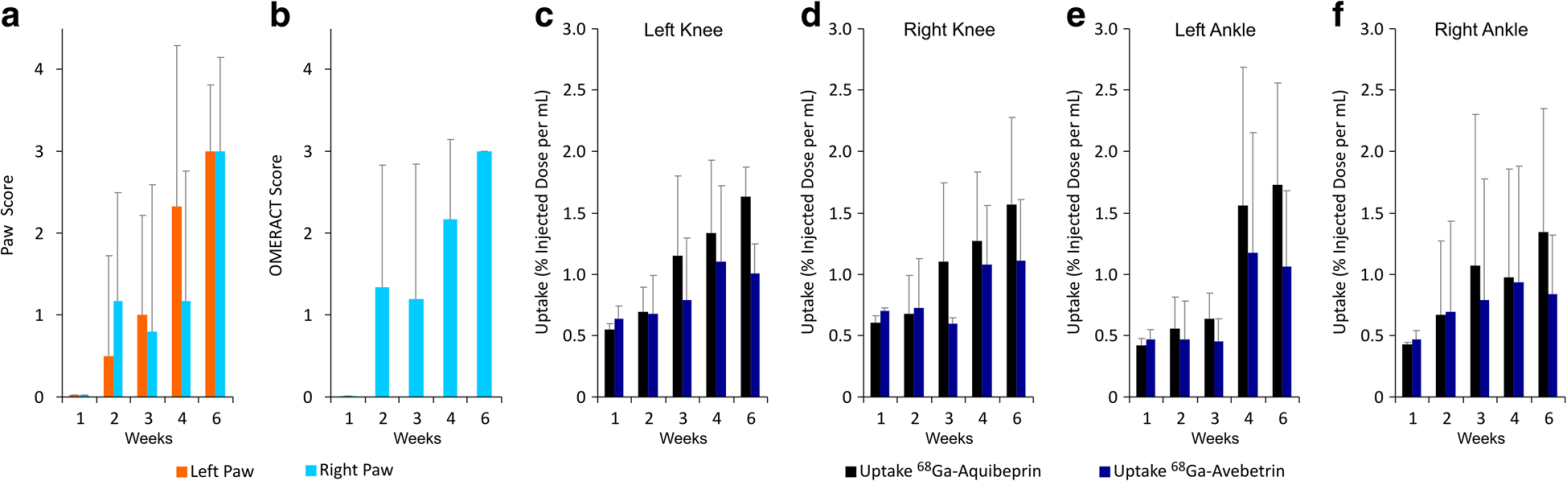
Clinical scores and PET uptakes of mice developing a collagen-induced arthritis (CIA). **a** Average clinical RA scores for hind limbs, ranging from 0 = no symptoms to 4 = severe arthritis. **b** Average MRI-based synovitis scores for right ankle, scores according to EULAR-OMERACT RAMRIS ranging from 0 = no symptoms to 3 = severe synovitis. **c**–**f** Region-of-interest (ROI) based uptakes of the PET imaging agents ^68^Ga-Aquibeprin (α5β1-integrin) and ^68^Ga-Avebetrin (αvβ3-integrin) in the left knee (**c**), right knee (**d**), left ankle (**e**), and right ankle (**f**). Data were generated for cohorts of 6 animals and are given as mean ± SD

In groups (*n* = 6) of collagen-induced arthritis (CIA) mice [32], the development of clinical RA symptoms was monitored by paw scoring (Fig. 1a). For the right hind paws, the extent of synovitis was additionally assessed by contrast-enhanced MRI and quantified according to the EULAR-OMERACT RAMRI scoring scheme (Fig. 1b). At the following day, two independent, subsequent PET scans were performed, utilizing the tracers ^68^Ga-Avebetrin for αvβ3-integrin and ^68^Ga-Aquibeprin for α5β1-integrin, respectively, and the uptakes in knee and ankle joints of the hind legs were quantified (Fig. 1c–f). Thereafter, the animals were sacrificed, selected joints were removed, decalcified, and embedded. Consecutive sections were subjected to MOVAT pentachrome staining for assessment of the extent of arthritic degeneration, as well as to β3- and α5-immunohistochemistry (IHC) for determination of the expression patterns of the target integrins.

### PET imaging of RA-associated expression of αvβ3- and α5β1-integrin

Figure 1 shows that the increase of the average clinical and MRI scores over time is accompanied with overall increasing integrin PET signals, which indicates that the integrin expression levels rise as the RA progresses. The consistently higher ^68^Ga-Aquibeprin uptakes suggest that the expression density of α5β1-integrin is higher, while a similar baseline uptake of both tracers (approx. 0.5 % ID/mL in week 1) indicates that the stronger α5β1 signal is not related to a higher unspecific retention in the tissue.

**Fig. 2:**
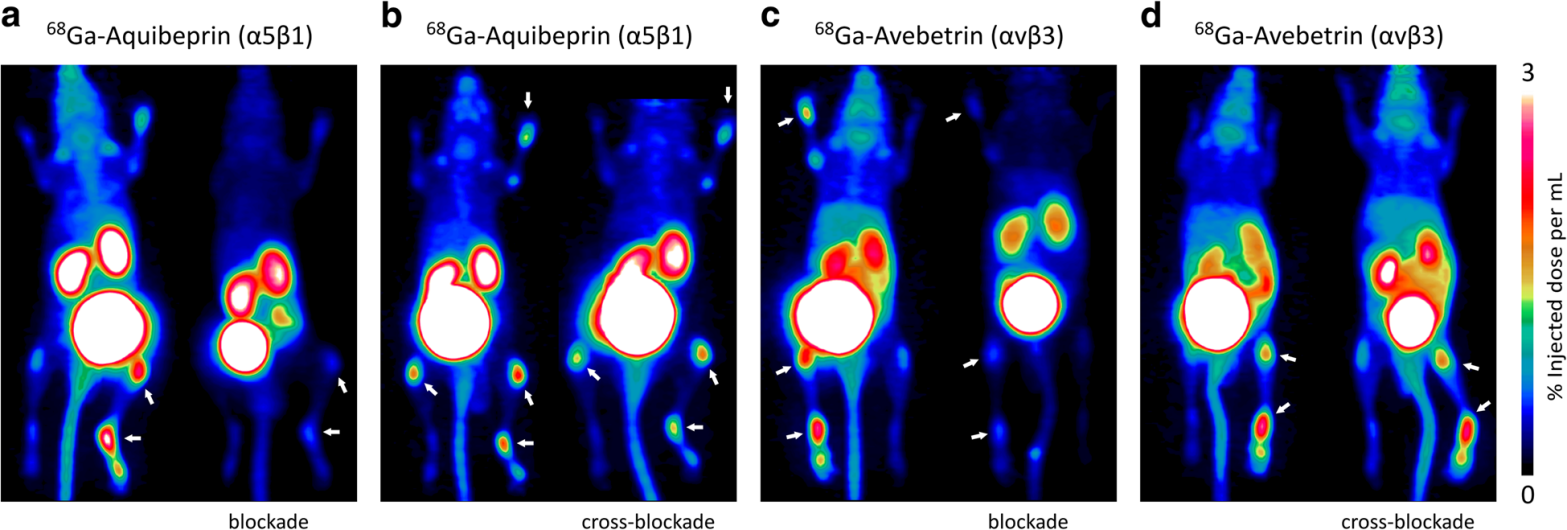
Subtype-specific integrin PET imaging in arthritic mice. **a**, **b** α5β1 integrin PET using ^68^Ga-Aquibeprin. **c**, **d** αvβ3 integrin PET using ^68^Ga-Avebetrin. Each of the images **a**–**d** shows two independent scans of the same mouse (different animals are shown in **a**–**d**). The arthritic joints are indicated by the arrows. The saturation of the receptor binding capacity by means of co-injection of a large dose (30 nmol) of unlabeled compound (blockade) resulted in a virtually complete reduction of the uptake in arthritic joints for both tracers, confirming the specificity of the PET imaging (**a**, **c**). The cross-blockade experiments confirmed the subtype-selectivity of α5β1- and αvβ3 integrin PET: The co-injection of 30 nmol Avebetrin did not substantially affect the α5β1 integrin imaging with ^68^Ga-Aquibeprin, while the minuscule reduction of the signal is likely related to a residual α5β1 activity (39 nM) of Avebetrin (**b**). The co-injection of 30 nmol Aquibeprin did not affect αvβ3 integrin imaging with ^68^Ga-Avebetrin (**d**)

In order to confirm the selectivity and specificity of both PET modalities for their respective integrin targets, we performed blockade and cross-blockade experiments for selected arthritic animals (Fig. 2). For both tracers, saturation of available receptors by co-injection of excess ligand resulted in a strong reduction of the PET signals in arthritic joints, proving that the observed signals are indeed caused by expression of the respective integrin and are not a result of unspecific accumulation mechanisms (Fig. 2a: control, 2.5; blockade, 0.5; Fig. 2c: control, 2.2; blockade, 0.5; uptakes given as average %ID/mL for joints marked with arrows). Additionally, independence of both signals was validated by saturation of the respective other receptor population by co-injection of an excess of the respective other ligands. Only slightly reduced signals for both combinations of imaging probe and blocking agent confirm the absence of cross-talk between the PET scans (Fig. 2b: control, 1.6; cross-blockade, 1.3; Fig. 2d: control, 2.0; blockade, 1.9; uptakes given as average %ID/mL for joints marked with arrows). Of note, the more pronounced reduction of the ^68^Ga-Aquibeprin signal is caused by a residual α5β1-integrin affinity of the co-injected Avebetrin (39 nM) [30]. This is too low to enable α5β1-integrin imaging with ^68^Ga-Avebetrin, but Avebetrin nevertheless competes to a small extent with ^68^Ga-Aquibeprin if administered in large amounts.

### IHC analysis of RA-associated α5β1-integrin levels

**Fig. 3:**
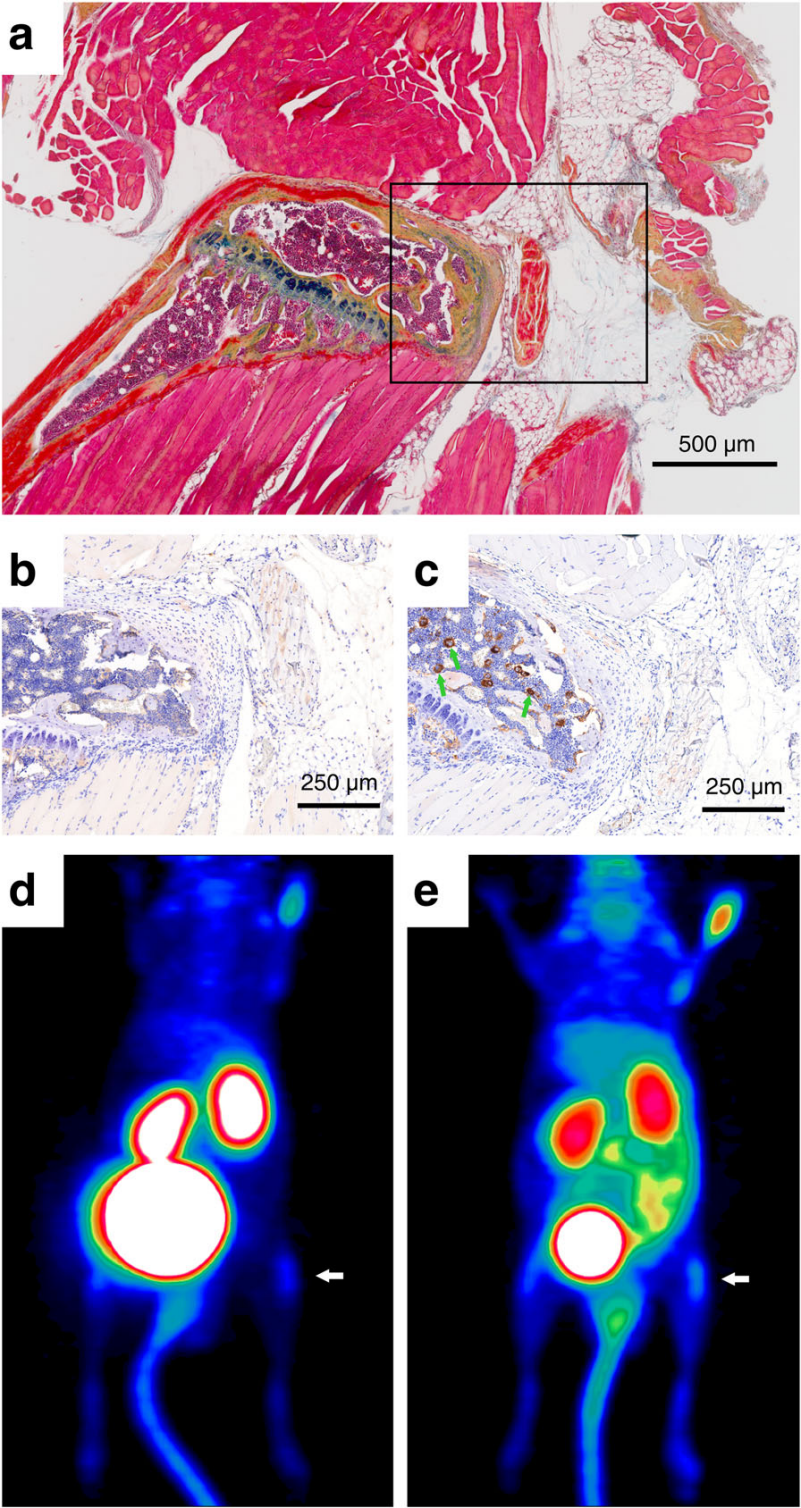
Integrin expression in non-arthritic joints. **a** MOVAT pentachrome staining of the right knee of a CIA mouse from week 2. The immunohistochemistry staining of consecutive slices for integrin α5 (**b**) and β3 (**c**), (depicted area corresponds to the inset frame in **a**). The same mouse was subjected to PET imaging of α5β1-integrin using 68Ga-Aquibeprin (**d**) and of αvβ3-integrin using ^68^Ga-Avebetrin (**e**), wherein the white arrow indicates the knee subjected to staining (**a**, **b**, **c**). No significant α5 expression was found (**b**), confirming the absence of α5β1. Focal expression of β3 (**c**, green arrows) originates from the expression of the platelet integrin αIIbβ3 by megakaryocytes (platelet progenitor cells); no significant β3 expression elsewhere confirms the absence of αvβ3. Consistently, both tracers show a low uptake in a non-arthritic knee. Color coding of μPET images (**d**, **e**) is similar to Fig. 2

The low PET background signal in the non-arthritic joints observed for both integrin tracers (see Fig. 3d, e, Fig. 1c–f) is in good agreement with the only focal weak IHC staining for the α5 and β3 proteins in the respective regions (Fig. 3b, c). Of note, α5-IHC is specific for α5β1 because α5 only dimerizes with β1. In contrast, the β3-specific antibody exhibits a cross-reactivity because β3 also dimerizes with the αIIb chain whereby the platelet integrin αIIbβ3 is formed. The focal β3 expression which is observed in the bone marrow (Fig. 3c) can be attributed to the presence of αIIbβ3-positive megakaryocytes and platelets, and thus, it does not indicate αvβ3-integrin presence.

**Fig. 4:**
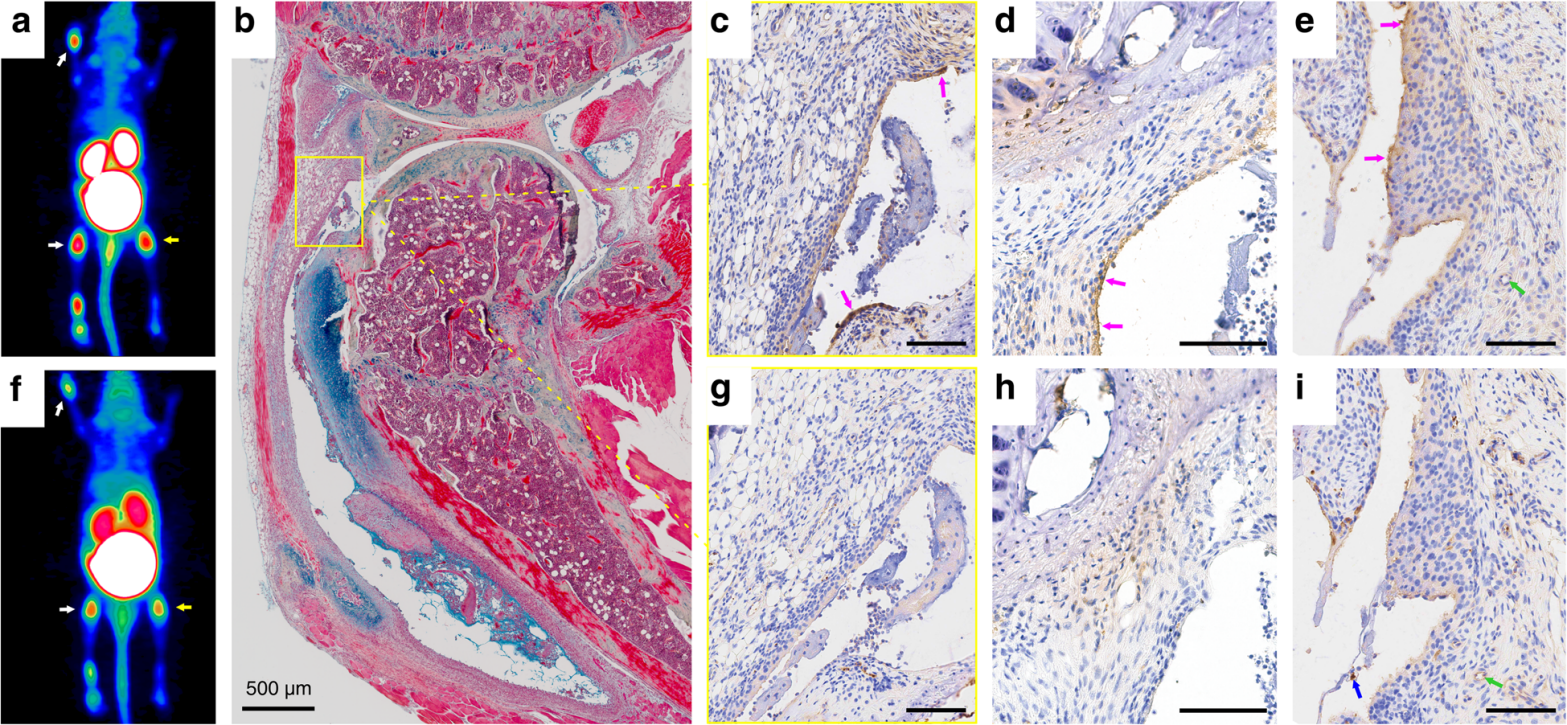
Integrin expression in arthritic joints. **a** ^68^Ga-Aquibeprin (α5β1-integrin) μPET for a DBA mouse from week 4. **b** MOVAT pentachrome staining of the right knee of the same animal used for μPET imaging (**a**, **f**). **c**–**e** α5-integrin immunohistochemistry (IHC). **f** ^68^Ga-Avebetrin (αvβ3-integrin) μPET for the same animal shown in **a**. **g**–**i** β3-integrin IHC. α5 and β3 IHC, respectively, are shown for consecutive slices of the right knee (**c**, **g**; area shown corresponds to the frame in **b**), left front paw (**d**, **h**), and left knee (**e**, **i**) of the same mouse used for PET (joints subjected to IHC are marked with arrows in **a** and **f**). In many areas, the synovial membrane shows multifocal α5 expression (indicated by pink arrows in **c**, **d**, and **e**) but virtually no presence of β3. Vascular endothelia (indicated by green arrows) show only a slight expression of α5 (**e**) but a moderate level of β3 (**i**). Furthermore, β3 is expressed by macrophages (**i**, blue arrow). In the respective arthritic joints, overall stronger α5 expression gives rise to a more intense signal for ^68^Ga-Aquibeprin (**a**) as compared to ^68^Ga-Avebetrin (**f**). Unless otherwise noted, all scale bars indicate 100 μm. Color coding of μPET images (**a**, **f**) is similar to Fig. 2

In contrast, the arthritic joints mostly show higher expression levels of α5-integrin than β3, as illustrated by a representative example (Fig. 4). The reactive synoviocytes present with a strong expression of α5-integrin, whereas β3-integrin is not identified within the synovial membrane. This is in agreement with the findings of other groups who reported that α5-integrin expression is increased in the synovial lining layer [21]. The advanced stages of RA show a moderate arthritis with mild cartilage and bone erosions as well as a hyperplastic synovitis associated with a strong α5-integrin expression (Fig. 4c–e), but still no β3-integrin staining (Fig. 4 g–i) within the synoviocytes. Throughout the RA development, high β3-integrin levels in macrophages and megakaryocytes were observed (Fig. 3c, Fig. 4i). The vascular endothelia within the vascular granulation tissue/pannus are also positive for α5-integrin (Fig. 4e), but only moderately express β3-integrin (Fig. 4i).

## Discussion

### Is α5β1-integrin a more specific biomarker for RA than αvβ3-integrin?

IHC (Fig. 4c–e) has shown that α5β1-integrin is predominantly found in the strongly proliferating synovial lining layer. This finding suggests that its expression is closely related to the specifically RA-related process of pannus formation and less so to a secondary process like angiogenesis. Hence, α5β1-integrin might evolve as a more specific biomarker for RA in joints than αvβ3-integrin, whose upregulation is less specifically related to RA development but more so to the accompanying angiogenesis. Hence, we assume that α5β1-integrin-directed diagnostic procedures for RA, such as molecular imaging, are less susceptible to interference with other biochemical processes involving angiogenesis than αvβ3-integrin-targeted approaches. At least, any RA-associated α5β1-integrin signals should be less related and/or superimposed by angiogenesis than those obtained for αvβ3-integrin, and, thus, be more specific for RA.

### Value of α5β1-integrin vs. αvβ3-integrin PET for prediction of RA progression

**Fig. 5:**
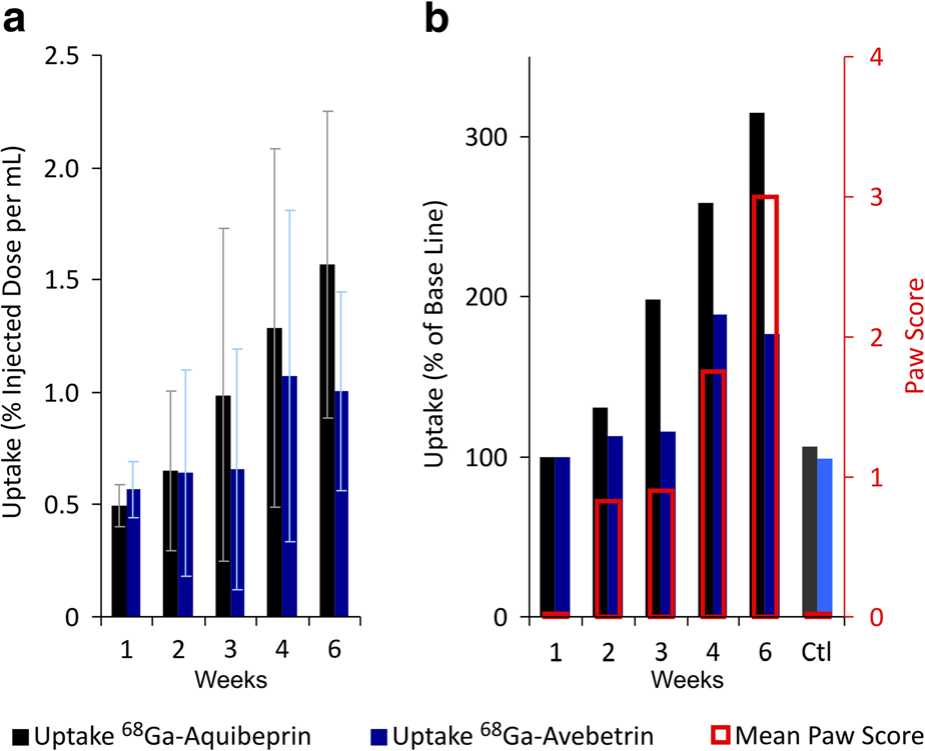
Growth of integrin PET signals over time and correlation with clinical RA indicators. **a** Uptake of 68Ga-Aquibeprin and ^68^Ga-Avebetrin in the arthritic ankles and knees (mean ± SD, *n* = 24), derived from PET data by an analysis of regions of interest (ROI). **b** Normalized uptakes (week 1 = 100%) and mean paw scores at the time of imaging. Error bars omitted for clarity; for data with error bars, see Fig. 1a and Fig. 5a. Ctl: data for non-arthritic mice at week 6 (control, *n* = 12)

Our data confirmed that the specific PET imaging of RA is feasible by targeting integrins αvβ3 and α5β1, partly corroborating the earlier preclinical and clinical studies with radiolabelled αvβ3-specific RGD peptides [25]. In this context, though, we recognize a substantial added value of α5β1-integrin PET. The evaluation of quantitative PET data reveals a somewhat lower baseline signal for α5β1 as compared to αvβ3 (Fig. 5a; see also Fig. 3d, e) which might be beneficial for clinical application because a lower background usually goes hand in hand with a higher sensitivity and thus increases the likelihood to detect earlier stages of RA by PET.

More importantly, our PET data indicate that during the progression of RA, the α5β1-integrin is upregulated earlier than αvβ3, raises steadily, and reaches higher levels than αvβ3 at later stages of the disease, which becomes even more apparent if the intensity of the PET signals is normalized to the respective baselines (Fig. 5b). Of note, the average paw scores indicate a mild but stable clinical picture of RA in weeks 2 and 3, which advances to a more severe state in week 4. This progress in week 4 is anticipated one week earlier by an α5β1-integrin expression, as the corresponding PET signal already increases in week 3. In contrast, during weeks 2–4, the αvβ3-signal is only rising to the same extent as the paw scores. This observation strongly supports our hypothesis that α5β1-integrin PET might have a value for prediction of RA development. Furthermore, the α5β1 signal keeps rising with the paw scores during the progression of RA (week 4 to week 6) and ultimately reaches > 300 % of the baseline. In contrast, the αvβ3 signal is leveling below 200 %. These data substantiate that α5β1-integrin PET is more suitable for RA imaging. The specificity of PET for RA was furthermore confirmed by control animals without RA induction, which showed only the baseline uptake at the end point (Fig. 5b).

### Prospects of α5β1-integrin PET to improve clinical management of RA

We have established that the α5β1-integrin PET enables the reliable localization of arthritic foci and furthermore anticipates RA progression. We conclude that this technique bears the potential to advance clinical management schemes of RA. Notably, the α5β1-integrin imaging adds a novel functional component to the diagnostic imaging portfolio, which could help to augment the established morphology-centered imaging methodology for RA and could provide additional information to medical practitioners suitable to achieve a higher precision in diagnostics, staging, and prognosis of RA.

Furthermore, we imagine an applicability for monitoring the therapy with DMARDs, which might be beneficial for patients because an early recognition of a lack of therapeutic effect might accelerate the decisions to terminate unsuccessful treatments and thus help to preserve the patients from the adverse effects of such medication. Furthermore, in light of the presumed predictive value of α5β1-integrin PET, it is reasonable to anticipate a substantial impact on RSO treatment planning, with the prospect of improving the life quality of RA patients who do not benefit from standard medication with DMARDs. Hence, we believe that α5β1-integrin PET will actually satisfy unmet clinical needs, demanding for immediate translation which is anticipated to result in routine clinical application.

## Conclusion

We compared the time course and location of the expression of the integrin subtypes α5β1 and αvβ3 in mouse models of rheumatoid arthritis (RA) by means of integrin-subtype specific PET imaging and immunohistochemistry. We found that α5β1 is predominantly located in the proliferating synovial lining layer, indicating that its expression might be closely related to a primary process of RA development in joints, namely, pannus formation. Hence, we assume that α5β1 is a more specific biomarker for RA development than αvβ3 whose expression is merely related to a secondary process, angiogenesis. Consequently, we found that during the progress of RA, the signals of α5β1-integrin targeted PET are rising earlier and stronger than the respective αvβ3-integrin PET signals. If the results of this study in murine models finally translate successfully to humans, α5β1-integrin PET could indeed offer the possibility for an early diagnosis of RA, possibly even before the manifestation of clinical symptoms. Since all RA treatment regimes benefit from an early start of therapy, α5β1-integrin PET might substantially improve the clinical management of RA and furthermore enhance the therapeutic outcome, particularly for patients with a hereditary risk.

## Data Availability

The datasets used and/or analyzed during the current study are available from the corresponding author on reasonable request.

## References

[1] Chaudhari K, Rizvi S, Syed BA (2016). Rheumatoid arthritis: current and future trends. Nat Rev Drug Discov..

[2] Lipsky PE (2000). Infliximab and methotrexate in the treatment of rheumatoid arthritis. N Engl J Med..

[3] Maini RN (1998). Therapeutic efficacy of multiple intravenous infusions of anti-tumor necrosis factor alpha monoclonal antibody combined with low-dose weekly methotrexate in rheumatoid arthritis. Arthritis Rheum..

[4] Feldmann M, Maini RN (2001). Anti-TNF alpha therapy of rheumatoid arthritis: what have we learned?. Annu Rev Immunol..

[5] Jones G, Nash P, Hall S (2017). Advances in rheumatoid arthritis. Med J Aust..

[6] Saag KG, Teng GG, Patkar NM (2017). American College of Rheumatology 2008 recommendations for the use of nonbiologic and biologic disease-modifying antirheumatic drugs in rheumatoid arthritis. Arthritis Rheum..

[7] Arnett FC, Edworthy SM, Bloch DA (1988). The American Rheumatism Association 1987 revised criteria for the classification of rheumatoid arthritis. Arthritis Rheum..

[8] Sommer OJ, Kladosek A, Weiler V, Czembirek H, Boeck M, Stiskal M (2005). Rheumatoid arthritis: a practical guide to state-of-the-art imaging, image interpretation, and clinical implications. Radiographics..

[9] Aletaha D, Neogi T, Silman AJ (2010). 2010 Rheumatoid Arthritis Classification Criteria. Arthritis Rheum.

[10] Rizzo C, Ceccarelli F, Gattamelata A (2013). Ultrasound in rheumatoid arthritis. Med Ultrason..

[11] Mäkinen H, Kaarela K, Huhtala H, Hannonen PJ, Korpela M, Sokka T (2012). Do the 2010 ACR/EULAR or ACR 1987 classification criteria predict erosive disease in early arthritis?. Ann Rheum Dis..

[12] Sugimoto H, Takeda A, Hyodo K (2000). Early-stage rheumatoid arthritis: prospective study of the effectiveness of MR imaging for diagnosis. Radiology..

[13] Sugimoto H, Takeda A, Masuyama J, Furuse M (1996). Early-stage rheumatoid arthritis: diagnostic accuracy of MR imaging. Radiology..

[14] McQueen FM (2008). The use of MRI in early RA. Rheumatology..

[15] Brown AK, Quinn MA, Karim Z (2006). Presence of significant synovitis in rheumatoid arthritis patients with disease-modifying antirheumatic drug-induced clinical remission: evidence from an imaging study may explain structural progression. Arthritis Rheum..

[16] Østergaard M, Dohn UM, Ejbjerg BJ, McQueen FM (2006). Ultrasonography and magnetic resonance imaging in early rheumatoid arthritis: recent advances. Curr Rheumatol Rep..

[17] Kubota K, Ito K, Morooka M (2011). FDG PET for rheumatoid arthritis: basic considerations and whole-body PET/CT. Ann N Y Acad Sci..

[18] Yamashita H, Kubota K, Mimori A (2014). Clinical value of whole-body PET/CT in patients with active rheumatic diseases. Arthritis Res Ther..

[19] Marrelli A, Cipriani P, Liakouli V (2011). Angiogenesis in rheumatoid arthritis: a disease specific process or a common response to chronic inflammation?. Autoimmun Rev..

[20] Paleolog EM (2002). Angiogenesis in rheumatoid arthritis. Arthritis Res..

[21] Lowin T, Straub RH (2011). Integrins and their ligands in rheumatoid arthritis. Arthritis Res Ther..

[22] Kapp TG, Rechenmacher F, Neubauer S (2017). A comprehensive evaluation of the activity and selectivity profile of ligands for RGD-binding integrins. Sci Rep..

[23] Aumailley M, Gurrath M, Müller G (1991). Arg-Gly-Asp constrained within cyclic pentapeptides – strong and selective inhibitors of cell-adhesion to vitronectin and laminin fragment-P1. FEBS Lett..

[24] Schottelius M, Laufer B, Kessler H, Wester HJ (2009). Ligands for mapping αvβ3-integrin expression in vivo. Acc Chem Res..

[25] Zhu Z, Yin Y, Zheng K (2014). Evaluation of synovial angiogenesis in patients with rheumatoid arthritis using ^68^Ga-PRGD2 PET/CT: a prospective proof-of-concept cohort study. Ann Rheum Dis..

[26] Neubauer S, Rechenmacher F, Beer AJ (2013). Selective imaging of the angiogenic relevant integrins α5β1 and αvβ3. Angew Chem Int Ed..

[27] Fassler R, Meyer M (1995). Consequences of lack of β1 integrin gene expression in mice. Genes Dev..

[28] Tanjore H, Zeisberg EM, Gerami-Naini B, Kalluri R (2007). β1 integrin expression on endothelial cells is required for angiogenesis but not for vasculogenesis. Dev Dyn..

[29] Atkinson SJ, Ellison TS, Steri V, Gould E, Robinson SD (2014). Redefining the role(s) of endothelial αvβ3-integrin in angiogenesis. Biochem Soc Trans..

[30] Notni J, Steiger K, Hoffmann F (2016). Complementary, Selective PET-imaging of integrin subtypes α_5_β_1_ and α_v_β_3_ using Ga-68-Aquibeprin and Ga-68-Avebetrin. J Nucl Med..

[31] Notni J, Steiger K, Hoffmann F (2016). Variation of specific activities of Ga-68-Aquibeprin and Ga-68-Avebetrin enables selective PET-imaging of different expression levels of integrins α_5_β_1_ and α_v_β_3_. J Nucl Med..

[32] Brand DD, Kary AL, Rosloniec EF (2007). Collagen-induced arthritis. Nat Protoc..

[33] Ostergaard M, Edmonds J, McQueen F (2005). An introduction to the EULAR-OMERACT rheumatoid arthritis MRI reference image atlas. Ann Rheum Dis..

[34] Sánchez-Crespo A, Andreo P, Larsson SA (2004). Positron flight in human tissues and its influence on PET image spatial resolution. Eur J Nucl Med Mol Imaging..

