# Correction to: In vivo imaging of early stages of rheumatoid arthritis by α5β1-integrin-targeted positron emission tomography

**DOI:** 10.1186/s13550-019-0582-x

**Published:** 2019-12-11

**Authors:** Johannes Notni, Florian T. Gassert, Katja Steiger, Peter Sommer, Wilko Weichert, Ernst J. Rummeny, Markus Schwaiger, Horst Kessler, Reinhard Meier, Melanie A. Kimm

**Affiliations:** 1 Institute of Pathology, Technische Universität München, Trogerstr, 18, 81675 Munich, Germany; 2 Department of Diagnostic and Interventional Radiology, Technische Universität München, Munich, Germany; 3 Department of Nuclear Medicine, Technische Universität München, Munich, Germany; 4 Institute for Advanced Study, Department of Chemistry, Technische Universität München, Garching, Germany; 5 German Cancer Consortium (DKTK), Partner Site Munich, Munich, Germany

**Correction to: EJNMMI Res (2019) 9:87**

**https://doi.org/10.1186/s13550-019-0541-6**

Following publication of the original article [1], the authors have reported an error in the ‘Histopathology’ (under ‘Materials and methods’) section of the article that compromises the reproducibility of the paper.

Namely, they have advised that the procedure for α5 integrin immunohistochemistry has not been reported correctly.

Unlike described in the paper, α5 IHC has been performed as follows:

“For alpha 5 integrin immunohistochemistry, a rabbit polyclonal antibody (LSBio, LS-B6179, diluted 1:7.500) was used. Immunohistochemistry was performed on a Leica BondRXm system (Leica, Wetzlar, Germany) using deparaffinization solution, pretreated with Epitope retrieval solution 2 (corresponding to EDTA buffer pH8) for 30 minutes. Antibody binding was detected with a polymer refine detection kit without post primary reagent and visualized with DAB as a dark brown precipitate.”

The authors apologize for this error.

## References

[1] Notni J (2019). In vivo imaging of early stages of rheumatoid arthritis by α5β1-integrin-targeted positron emission tomography. EJNMMI Res.

